# Estimating Child Mortality at the Sub-national Level in Papua New Guinea: Evidence From the Integrated Health and Demographic Surveillance System

**DOI:** 10.3389/fpubh.2021.723252

**Published:** 2022-01-27

**Authors:** Bang Nguyen Pham, Rebecca Bogarobu Emori, Tam Ha, Anne-Maree Parrish, Anthony D. Okely

**Affiliations:** ^1^Population Health and Demography Unit, Papua New Guinea Institute of Medical Research, Goroka, Papua New Guinea; ^2^School of Health and Society, University of Wollongong, Wollongong, NSW, Australia; ^3^Early Start and Illawarra Health and Medical Research Institute, University of Wollongong, Wollongong, NSW, Australia

**Keywords:** child mortality, surveillance system, Papua New Guinea, direct estimations, indirect estimation

## Abstract

**Background:**

Child mortality is an important indication of an effective public health system. Data sources available for the estimation of child mortality in Papua New Guinea (PNG) are limited.

**Objective:**

The objective of this study was to provide child mortality estimates at the sub-national level in PNG using new data from the integrated Health and Demographic Surveillance System (iHDSS).

**Method:**

Using direct estimation and indirect estimation methods, household vital statistics and maternal birth history data were analysed to estimate three key child health indicators: Under 5 Mortality Rate (U5MR), Infant Mortality Rate (IMR) and Neonatal Mortality Rate (NMR) for the period 2014–2017. Differentials of estimates were evaluated by comparing the mean relative differences between the two methods.

**Results:**

The direct estimations showed U5MR of 93, IMR of 51 and NMR of 34 per 1000 live births for all the sites in the period 2014–2017. The indirect estimations reported an U5MR of 105 and IMR of 67 per 1000 live births for all the sites in 2014. The mean relative differences in U5MR and IMR estimates between the two methods were 3 and 24 percentage points, respectively. U5MR estimates varied across the surveillance sites, with the highest level observed in Hela Province (136), and followed by Eastern Highlands (122), Madang (105), and Central (42).

**Discussion:**

The indirect estimations showed higher estimates for U5MR and IMR than the direct estimations. The differentials between IMR estimates were larger than between U5MR estimates, implying the U5MR estimates are more reliable than IMR estimates. The variations in child mortality estimates between provinces highlight the impact of contextual factors on child mortality. The high U5MR estimates were likely associated with inequality in socioeconomic development, limited access to healthcare services, and a result of the measles outbreaks that occurred in the highlands region from 2014-2017.

**Conclusion:**

The iHDSS has provided reliable data for the direct and indirect estimations of child mortality at the sub-national level. This data source is complementary to the existing national data sources for monitoring and reporting child mortality in PNG.

## Introduction

In 1990, approximately 12 million children died globally before they reached their fifth birthday (1). In 2000, the United Nations' Millennium Development Goal 4 (MDG 4) set out a target to reduce child mortality by two-thirds by 2015 (2). Globally, significant progress in the improvement of child health has been made. There has been a 50% decline in child mortality, from 12.7 million in the 1990's to 6.3 million in 2013 (3). However, to date only 31 countries reported having met the MDG targets for child mortality (4, 5). Addressing high child mortality remains a global health priority in the United Nations' Sustainable Development Goals (SDG). SDG 3 target 3.2 to reduce neonatal mortality to 12 per 1,000 live births and under-5 mortality to 25 per 1,000 live births by 2030 (6).

Child mortality is an important indicator of global public health. To monitor progress towards the SDG 3, it is vital to have accurate estimates of under-five mortality rate (U5MR), infant mortality rate (IMR) and neonatal mortality rate (NMR) at the global, national and sub-national levels. National vital and civil registration systems are often referred to as reliable data sources for the estimation of child mortality, based on the complete registration of births and deaths in the population (7, 8). However, these systems may not exist or be fully functioning in many low- and middle income countries (LMICs), where most childhood deaths occur (8). Hence, for these countries, child mortality estimates heavily rely on cross-sectional household survey data. The most common data sources available at the national level are Demographic and Health Surveys (DHS) and National Censuses, in which maternal birth history data are retrospectively recorded either in full or in summary and may be used to estimate child mortality (9).

Estimations of child mortality indicators are widely used by governments and international development partners to monitor and report the progress of international development agendas such as SDGs to inform public policy and national socioeconomic development programs for improving the health and wellbeing of children (10). Thus, lack of data for reliable estimations of child mortality at the national and sub-national levels could lead to ineffective child health policy, affecting childhood communicable diseases preventive measures and child health protection policies (11). Accurate estimation of child mortality largely depends on two main factors: the data quality and the method used for estimation (12).

Although efforts have been made to improve the health of children in the period 2000s-2010s, child mortality is still reportedly high in Papua New Guinea (PNG) and PNG is amongst countries with the highest child mortality rates in the Pacific region (13). Like many LMICs, PNG did not achieve the MDG 4 (14). The lack of updated and reliable data rendered the PNG Government unable to fully report the country's progress and achievement of the MDG 4 to the United Nations in 2015 (14). The PNG Government through its Medium Term Development Plan 2018–2022 (MTDP) has set specific targets to improve child survival in line with the SDG 3. According to this plan, the country aims to reduce the IMR and U5MR to 17 and 20 per 1,000 live births by 2022, respectively (15, 16).

PNG faces challenges in tracking and reporting the country's progress toward achieving the international and national development targets due to lack of reliable data sources and adequate human and financial resources (17). While national vital and civil registration systems are generally known to be reliable data sources for the ‘direct estimation' of child mortality, these systems are not fully developed and functioning in PNG in order to provide a complete data series on birth and death statistics (18). The DHS and National Censuses have been used over the last four decades 1990–2020's as the only national data sources available in PNG for estimation of child mortality (19, 20). The most recent data from the DHS 2016 provides U5MR of 49, IMR of 33 and NMR of 20 deaths per 1,000 live births (21). However, the child mortality estimates based on these data sources have never been validated against other data sources in terms of accuracy and reliability.

Bauze et al. (22) assessed the quality of data from the DHS 2006 and they suggested that U5MR estimation should be conducted at the sub-national level by further exploring alternative options of birth and death surveillance data. These measures could include face to face data collection at the village level, particularly in rural and remote regions where child mortality estimates are likely to be higher and under reported.

Given the increasing importance of child mortality data, this study was conducted to estimate child mortality at the sub-national level in PNG, using new data from the integrated Health and Demographic Surveillance System (iHDSS), operated by the Papua New Guinea Institute of Medical Research (PNGIMR). PNGIMR's iHDSS is one of 49 surveillance centres of the International Network for the Demographic Evaluation of Populations and their Health (INDEPTH), a global surveillance network that collects high-quality vital registration information in diverse settings of LMICs by carrying out a series of mortality and morbidity surveillance of the defined populations, in which the fertility and mortality are integrated as part of the household socioeconomic data. These ‘ground truth' data series can be used to provide new insights into the child mortality estimates in different demographic and epidemiological settings (23). Little is known about child mortality estimations using data from the iHDSS, especially estimates at the sub-national level.

The objective of this study was to provide child mortality estimates at the sub-national level in PNG using new data from the integrated Health and Demographic Surveillance System (iHDSS). Child mortality direct and indirect estimation methods were used in this study to address four specific research questions: (i) What are the child mortality estimates using the direct estimation method? (ii) What are the child mortality estimates using the indirect estimation method? (iii) How do the child mortality estimates produced by the direct and indirect estimation methods vary? and (iv) How do child mortality estimates vary between provinces?

## Materials and Methods

### Data Source

This study used the PNGIMR's iHDSS data collected in the period 2014–2017, the latest time period when appropriate data for child mortality estimation were collected. The iHDSS was designed as a population-based longitudinal cohort study. The overall purpose of the system was to provide a reliable and up-to-date data series for monitoring the implementation of socioeconomic development programmes and healthcare interventions at the sub-national level in PNG. The iHDSS methodology has been described elsewhere (24, 25).

The geographic coverage of the iHDSS consisted of four rural sites, namely Asaro in Eastern Highlands Province (EHP), Hides in Hela Province, Hiri in Central Province, and Karkar in Madang Province, representing three out of the four geographical regions of PNG: the Highlands, the Southern, the Momase, respectively (no surveillance site in the Islands region in the period of this study) (26). These surveillance sites were selected based on the developmental history and surveillance experience of PNGIMR in consultation with the PNG Government agencies and stakeholders.

**Figure 1:**
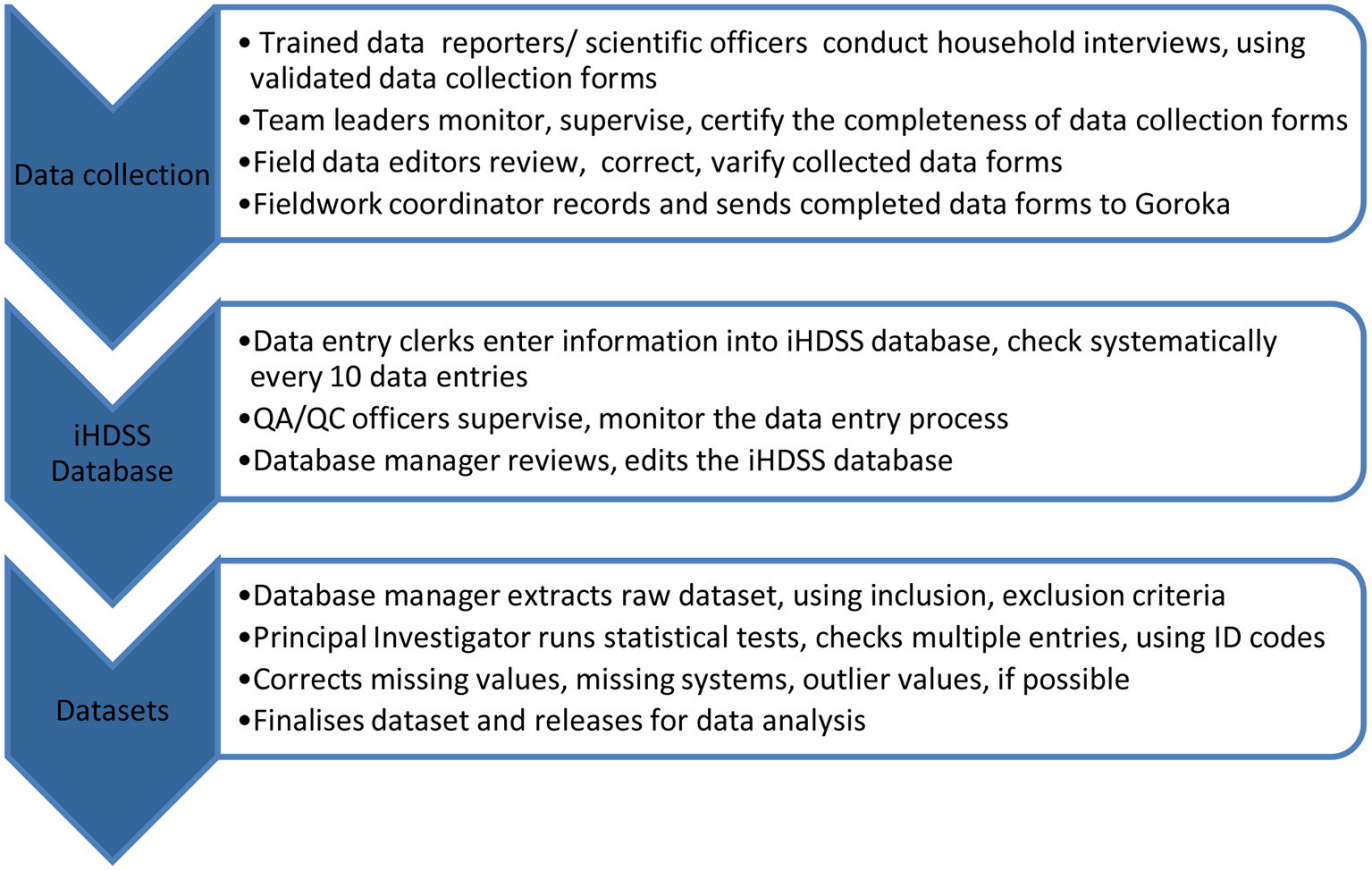
Quality assurance and quality control procedures, PNG IMR's iHDSS, 2011-2017.

As of the end of 2017, the population coverage of the iHDSS was about 54,000 people, equivalent to 1% of the total population of PNG in 2011 when the system was set up. Data collection was conducted by the iHDSS teams via household interviews, using various data collection tools, which were translated from English into *Tok-Pisin*, the most common language spoken by Papua New Guineans. New datasets were released twice a year for data analysis and reporting purposes (26). Data quality assurance and quality control procedures are presented in Figure 1.

### Settings

Table 1 shows the overall socioeconomic description of the four surveillance sites. Asaro and Hides sites are in the highlands region while Hiri and Karkar sites are both in coastal areas.

**Table 1:** Overall description of socioeconomic status of surveillance site, PNGIMR's iHDSS, 2016.

**Province**	**EHP**	**Hela**	**Central**	**Madang**
Region	Highlands	Highlands	Southern	Momase
Surveillance site	Asaro	Hides	Hiri	Karkar
Location	40 km, northeast of Goroka township	45 km south of Tari township	45 km west of Port Moresby	Island 30 km from Madang township
Main industry	Coffee, sweet potatoes	Coffee, hunting, Liquefied Natural Gas	Fishery, hunting, Liquefied Natural Gas	Fishery, plantation (cocoa, coconut)
Accessibility	Road and airline	Road and airline	Road	Sea
Year of operation	2011-current	2011–2017	2011-current	2011–2015
Surveillance population	10,000	13,000	12,000	19,000
Surveillance household	2,000	2,500	2,000	3,000
Primary public health facility	Asaro health centre, Uritoka health clinic, Tafeto health clinic	Malanda health centre, Para health clinic	Porebada, Papa, and Lealea health clinics	Gaubin hospital, Miak, Wadau, and Kuburne health clinics
Laboratory service	N/A	Goroka Lab	N/A	Madang Lab

Asaro site is located approximately 40–45 km northeast of Goroka. Asaro is primarily a farming and agricultural production area. Coffee and sweet potatoes (*kaukau*) are the main cash crop. Major languages spoken by people living in Asaro are *Tokples, Gahuku, Siane* and *Dano/Tokano*, apart from *Pidgin* that is also regularly spoken. There are three primary health facilities where local people in the site can have access to basic health services.

Geographically, the Hides site is very remote and difficult to access. Tribal cultural norms and practises are an integral part of the local people's lives and have created a complex society. People live in clans and sub-clans, and maintain a traditional tribal lifestyle. Most of the houses are built using bush materials and there are very few semi-permanent buildings. The main *Tokples* language spoken is *Huli*. Hides site is also home to the Komo Airfield.

Hiri site is located in Central Province, approximately 30–40 km west of Port Moresby, the National Capital of PNG. The iHDSS covers four coastal villages i.e., *Porebada, Boera, Papa* and *Lealea*. Each village has its own health clinic. Most inhabitants are either *Motu* or *Koitabu* speakers. Hiri site can be reached by road in <1 h from Port Moresby.

Karkar district is a volcanic island located 30 km off the PNG coast in the Bismarck Sea. The island's soil is known for its fertility and the large plantations produce the island's main exports of cocoa and coconut and provide a large amount of the local employment opportunities. Inhabitants of the island come from one of two language groups: *Waskia* in the North half of the island and *Takia* in the South. Karkar site was closed at the end of 2015 due to staff safety issues.

### Estimating Child Mortality

The iHDSS adopted a life-circle approach to capture key demographic events such as births, deaths and migration at the household level, as well as individual health data of the defined populations including women of reproductive age 15–49 years, and children under 5 years of age. Hence, these data from the iHDSS can be used for estimations of child mortality indicators. Two estimation methods were used in this study: (i) the direct estimation method is based on household demographic change data including birth and death records; and (ii) the indirect estimation method uses maternal birth history data. A summary of these two estimation methods are presented in Table 2. Comparing and contrasting of child mortality estimates between the two methods could also evaluate the reliability and accuracy of the estimates.

**Table 2:** Summary of direct and indirect estimation methods used for estimation of child mortality in Papua New Guinea, PNGIMR's iHDSS 2014–2017.

	**Direct estimation method**	**Indirect estimation method**
Estimation/calculation method	(Total child deaths)/(Total live births) ×1,000	Brass W method (model life tables)
Data component	Household socioeconomic	Women of aged 15–49 years
Data collection tool	Household Update Book	Women of reproductive age Questionnaire
Data module	Household demographic change: births, deaths, migration	Child mortality
Variables used	Date of birth, date of death, age at death, total numbers of live births, CU5, infant and neonatal deaths in a given period of time	Women' birth history: Numbers of child ever born, child survived, child dead, maternal age
Data collector	Village-based data collectors	National scientific officers
Data collection period	July-December 2014 July-December 2017	July-December 2015
Child mortality indicators reported	NMR, IMR, and U5MR	IMR and U5MR
Year of estimate	Each year in the period 2014–2017	2014 only

This study reports on three key child mortality indicators: (i) NMR is the proportion of children dying within the first month of life (age at death is between 0 and 27 days); (ii) IMR is the proportion of children dying before their first birthday (age at death is between 0 and 11 months; (iii) U5MR is the proportion of children dying before their fifth birthday (age at death is between 0 and 4 years). These child mortality rates are expressed per 1,000 live births. Stillbirth rates are not included in these analyses.

### Direct Estimation

In the direct estimation, child mortality rates were calculated by dividing the total number of child deaths by the total number live births recorded in a given period of time i.e., one year. This method involves birth and death data, extracted from the iHDSS household socioeconomic data component. Household Update Book (HUB) was used to record household identification information, list of all household members, and their identification information such as name, sex, date of birth, date of death, and date of migration in and out of the villages. Data used in this study were mostly from the data module on household demographic changes, including birth and death data. Main variables were used including the number of children born in the household, the child's date of birth (day, month and year), the age of the child at the time of the visit (in days, months or years), and sex of the child. Death statistics included the number of deaths in the household, the date of their death (date, month, and year) and their age at death (in years, months and days).

This information was collected by village-based data collectors. With the social network, local background and knowledge, data collectors had an advantage when collecting these data from households in their localities. Data collectors were trained to use the HUB and basic interview skills prior to their visits to the households. They asked household members, mostly household heads to provide information about the births and deaths that occurred in the household since the last visit. Two rounds of HUB data collection were conducted in July-December 2014 (in all four provinces), and in July-December 2017 (for three provinces, except Madang).

The HUB dataset was extracted from the iHDSS database for direct estimation of child mortality rates. The information on “date of birth” and “date of death” were used to calculate “age at death” of deceased children in the households, which allowed checking for internal consistency across information provided by the respondents. The “age at death” data were used to classify child deaths into three groups: neonatal mortality (0–27 days), infant mortality (0–11 months) and under 5 mortality (0–4 years). Three child mortality indicators: NMR, IMR and U5MR were estimated for each surveillance site and for all sites for 2014, 2015, 2016, and 2017, and for the entire period 2014–2017. Findings are presented in Table 3.

**Table 3:** Direct estimation of under 5 mortality rate, infant mortality rate and neonatal mortality rate using birth and death data from household update book, PNGIMR's iHDSS, 2014–2017.

**Year of estimate**	**Child morality estimates**	**Central**	**EHP**	**Madang**	**Hela**	**All provinces**
2014	Total live births	254	204	153	243	854
	Number of children under 5 deaths	10	30	16	31	87
	Number of infant deaths	4	16	8	17	45
	Number of neonatal deaths	3	10	7	12	32
	U5MR estimate (per 1,000 live birth)	39.37	147.06	104.58	127.57	101.87
	IMR estimate (per 1,000 live births)	15.75	78.43	52.29	69.96	52.69
	NMR estimate (per 1,000 live birth)	11.81	49.02	45.75	49.38	37.47
2015	Total live births	302	220	NA	217	739
	Number of children under 5 deaths	11	26	NA	32	69
	Number of infant deaths	5	18	NA	16	39
	Number of neonatal deaths	5	9	NA	11	25
	U5MR estimate (per 1,000 live birth)	36.42	118.18	NA	147.47	93.37
	IMR estimate (per 1,000 live births)	16.56	81.82	NA	73.73	52.77
	NMR estimate (per 1,000 live birth)	16.56	40.91	NA	50.69	33.83
2016	Total live births	328	296	NA	98	722
	Number of children under 5 deaths	15	33	NA	14	62
	Number of infant deaths	6	21	NA	8	35
	Number of neonatal deaths	6	12	NA	5	23
	U5MR estimate (per 1,000 live birth)	45.73	111.49	NA	142.86	85.87
	IMR estimate (per 1,000 live births)	18.29	70.95	NA	81.63	48.48
	NMR estimate (per 1,000 live birth)	18.29	40.54	NA	51.02	31.86
2017	Total live births	152	128	NA	57	337
	Number of children under 5 deaths	7	15	NA	7	29
	Number of infant deaths	3	9	NA	4	16
	Number of neonatal deaths	3	5	NA	3	11
	U5MR estimate (per 1,000 live birth)	46.05	117.19	NA	122.81	86.05
	IMR estimate (per 1,000 live births)	19.74	70.31	NA	70.18	47.48
	NMR estimate (per 1,000 live birth)	19.74	39.06	NA	52.63	32.64
2014-2017	Total live births	1,036	848	153	615	2,652
	Number of children under 5 deaths	43	104	16	84	247
	Number of infant deaths	18	64	8	45	135
	Number of neonatal deaths	17	36	7	31	91
	U5MR estimate (per 1,000 live birth)	41.51	122.64	104.58	136.59	93.14
	IMR estimate (per 1,000 live births)	17.37	75.47	52.29	73.17	50.90
	NMR estimate (per 1,000 live birth)	16.41	42.45	45.75	50.41	34.31

### Indirect Estimation

The indirect estimation method for child mortality was first introduced by Brass William (27). This method summarises birth history data using a model life table (mathematical models of the variation of mortality with age) to estimate proportions of child death in specific maternal age groups for the years prior to the interview time. This is achieved by taking into account the approximate length of exposure of children to the risk of dying, and assuming a particular model age pattern of mortality (28, 29).

The dataset used for indirect estimation of child mortality was extracted primarily from the women of reproductive age 15–49 years data component of the iHDSS database. These data were collected by the PNGIMR's iHDSS national scientific officers using the individual questionnaire on Women of Reproductive Age, 15–49 years in the period from July-December 2015. This questionnaire was designed by the iHDSS team in 2014, based on various existing data collection tools in use across international organisations such as the INDEPTH, the United Nations' Multi-indicator Cluster Survey (MICS), and the Measure Evaluation's DHS. The questionnaire comprises eight data modules: Household identification information, Women's background, Marriage and family, Sexual behaviour, Domestic violence, Child mortality, Unmet need for contraception, and Maternal and newborn health.

Data from the child mortality component of the iHDSS database were used in the current study. In this data module, women were asked questions about their birth history, including maternal age, numbers of son/daughter living at home, and numbers of sons/daughters living elsewhere, and numbers of boys/girls born alive but died later. Based on this information, new variables on total numbers of live births, children surviving, and children deceased were created. These new variables were used to calculate a mean of total live births and a mean of total children surviving per woman, in order to estimate total live births, total children surviving, and total children deceased for each woman who participated in the study. Based on these estimates, the proportion of children, who had died was calculated for 5-years maternal age groups i.e., 15–19, 20–24, 25–29, 30–34, 35–39, 40–44, and 45–49 years, in each province and for all provinces.

This estimation process and data on U5MR and IMR estimates are shown in Table 4. U5MR and IMR estimates are obtained by converting the proportions of children dead, which was obtained from the step (vii) into probabilities of children dying for 2014, the year prior to the data collection. Descriptive statistical data analyses were conducted using Statistics Package of Social Science (SPSS version 20.0).

**Table 4:** Estimation of under 5 mortality and infant mortality rates in 2014, Brass indirect estimation method, women aged 15–49 birth history data, PNGIMR's iHDSS, 2015.

**Province**	**Woman age group**	**No. of women**	**Mean of total live births**	**Mean of total children surviving**	**Estimated total live birth**	**Estimated total children surviving**	**Estimated total children dead**	**Proportion of children dead**
		**(i)**	**(ii)**	**(iii)**	(iv)= (i)*(ii)	(v)= (i)*(iii)	**(vi) = (iv)−(v)**	**(vii) = (vi)/(iv)**
Central	15–19	455	1.52	1.49	692	678	14	0.020
	20–24	407	1.58	1.50	642	611	31	0.049
	25–29	387	2.84	2.70	1,101	1,045	56	0.051
	30–34	307	3.69	3.54	1,134	1,087	47	0.042
	35–39	287	4.25	3.67	1,220	1,053	166	0.136
	40–44	246	4.68	3.84	1,151	944	207	0.180
	44–49	174	4.90	4.02	853	699	153	0.180
EHP	15–19	343	2.00	1.80	686	617	69	0.100
	20–24	262	2.35	1.93	615	507	108	0.176
	25–29	266	2.96	2.62	789	696	92	0.117
	30–34	289	4.64	3.90	1,340	1,127	213	0.159
	35–39	194	4.79	3.90	928	757	171	0.184
	40–44	285	5.63	4.47	1,606	1,274	331	0.206
	44–49	167	5.82	4.93	973	824	149	0.153
Madang	15–19	313	1.60	1.50	501	470	31	0.063
	20–24	235	1.81	1.60	426	376	50	0.117
	25–29	193	2.57	2.28	496	441	56	0.112
	30–34	177	3.49	3.11	617	551	66	0.108
	35–39	169	4.35	4.07	735	687	48	0.065
	40–44	139	5.42	4.94	754	687	67	0.089
	44–49	82	5.80	5.03	476	412	63	0.133
Hela	15–19	66	1.00	1.00	66	66	0	N/A
	20–24	25	2.00	1.47	50	37	13	0.265
	25–29	32	2.33	2.24	75	72	3	0.041
	30–34	23	2.25	2.20	52	51	1	N/A
	35–39	9	4.67	3.89	42	35	7	0.167
	40–44	15	4.20	3.54	63	53	10	0.158
	44–49	7	5.80	3.29	41	23	18	0.433
All provinces	15–19	1,177	1.50	1.40	1,766	1,648	118	0.067
	20–24	929	1.93	1.62	1,791	1,507	283	0.158
	25–29	878	2.84	2.37	2,494	2,083	411	0.165
	30–34	796	3.98	3.56	3,166	2,834	332	0.105
	35–39	659	4.42	3.83	2,915	2,526	389	0.133
	40–44	685	5.30	4.30	3,632	2,946	686	0.189
	44–49	430	5.27	4.48	2,264	1,927	337	0.149
	Total	5,554	4.00	3.17	22,216	17,615	4,601	0.207

### Comparison of Child Mortality Estimates Produced by Direct and Indirect Estimations

To show the differences of child mortality estimates, mean relative differences between U5MR estimates and IMR estimates derived from the direct and indirect estimation methods were calculated for each province (except for Madang) and for all provinces. The relative difference *d* between these two estimation methods was calculated using the formula below:


$$\begin{array}{l} d_{i}=\;\frac{x_{{i,indirect}^{\;-\;}}x_{i,\;direct}}{\frac{1}{2}\times \left(x_{i,indirect}+x_{i,direct}\right)} \end{array}$$

Where, *x I, indirect* and *x I, direct* are the estimated child mortality rates derived from the indirect method and the direct method, respectively, for year *i* in a population. This method has been discussed elsewhere (23).

U5MR and IMR estimates for the calendar year 2014 obtained from the direct estimations were included in the calculation of paired means of relative differences between indirect and direct estimates to ensure the estimated referencing periods between the two methods corresponded. The paired means of relative differences indicate whether the indirect estimates are measurably different from the corresponding direct estimates. Data are presented in Table 5.

**Table 5:** Mean relative differences (reported as percent points) between indirect and direct estimations for IMR and U5MR estimates in 2014, PNGIMR's iHDSS, 2015.

**Province**	**Child mortality**	**Indirect estimate**	**Direct estimate**	**Indirect −Direct**	**Indirect + Direct**	**Mean (indirect + direct)**	**Mean relative difference**
		**(1)**	**(2)**	**(3) = (2) – (1)**	**(4) = (2) + (1)**	**(5) = [(1) + (2)]/2**	**(6) = (3)/(5)**
Central	IMR	19.74	15.75	3.99	35.49	17.74	0.225
	U5MR	41.80	39.37	2.43	81.17	40.59	0.060
EHP	IMR	100.00	78.43	21.57	178.43	89.22	0.242
	U5MR	158.82	147.06	11.76	305.88	152.94	0.077
Madang	IMR	63.00	52.29	10.71	115.29	57.65	0.186
	U5MR	108.00	104.58	3.42	212.58	106.29	0.032
Hela	IMR	N/A	69.96	N/A	N/A	N/A	N/A
	U5MR	N/A	127.57	N/A	N/A	N/A	N/A
All provinces	IMR	67.00	52.69	14.31	119.69	59.85	0.239
	U5MR	104.99	101.87	3.12	206.86	103.43	0.030

## Results

Table 3 shows the direct estimates of U5MR, IMR and NMR in the period 2014-2017, based on the numbers of total live births and child deaths recorded in the iHDSS database. U5MR estimates in EHP declined in this period, from the level of 147 per 1,000 live births in 2014 to 111 in 2016. A similar trend was also observed in Hela, where U5MR estimates declined from 147 per 1,000 live births in 2015 to 123 in 2017. In contrast, U5MR in Central remained low with 42 per 1,000 live births during the period 2014–2017. Unlike the varied U5MR estimates, IMR estimates were relatively stable with 50 deaths per 1,000 live births for all sites from 2014~2017, with highest estimates observed in EHP and Hela (~70–80 per 1,000 live births), and lowest in Central (~15~20 per 1,000 live births). Similarly, NMR estimates were ~30–35 per 1,000 live births for all sites from 2014 to 2017, but the highest level was estimated for Hela (30), followed by EHP and Madang (31–36), and lowest in Central (10–20).

Table 4 illustrates the process of indirect estimation of U5MR and IMR using Brass indirect estimation method. Birth history data of 5,554 women aged 15–49, including 22,216 live births, 17,615 children surviving, and 4,601 children dead were included in the estimation. The proportions of child deaths in two maternal age groups: 15–19 and 30–34 years were 0.067 and 0.105, respectively. Hence, the IMR estimate was approximately 67 deaths per 1,000 live births and the U5MR estimate was approximately 105 child deaths per 1000 live births for all provinces. The IMR estimate was lowest in Central Province (20), followed by Madang (63), and the highest estimate in Eastern Highlands (100). The U5MR estimates were highest in EHP (159), followed by Madang (108), and lowest in Central (42) (data of Hela were ineligible for estimation since small numbers were reported). These estimates were referred to mid-2014, the year prior to the data collection.

Table 5 shows the mean relative differences of IMR and U5MR estimates calculated from the indirect and direct estimations. NMR rates were not included in this analysis as the data were only available from the direct estimation. The indirect estimation provided higher IMR estimates than the direct estimation by 24 percentage points for the IMR estimate for all provinces. However, the U5MR direct and indirect estimates were relatively consistent, varying by 3 percentage points for all provinces. Further examining the mean relative differences of IMR and U5MR estimates at the provincial level, the data showed that the IMR and U5MR estimates were most reliable in Madang, where the mean relative differences of IMR and U5MR estimates were minimum among provinces, at 18 and 3 percentage points, respectively.

## Discussion

To our knowledge this is the first study to provide an update on child mortality estimates in PNG at the sub-national level using the data from the iHDSS. National data sources available for child mortality estimations were reviewed to highlight gaps in analysing and monitoring child mortality at the sub-national levels.

### Comparison Between Direct and Indirect Child Mortality Estimates

The use of direct and indirect estimation methods strengthened the rigour of the research method of this study. Comparison between these methods highlighted the consistency and the differences between the methods. This comparison sheds light on the reliability of the child mortality estimates based on the vital statistics of households for direct estimation and maternal birth history data for indirect estimation. This approach is a methodological strength of our study, allowing assessment of the reliability of the surveillance data by comparing the two data sets to determine the accuracy of child mortality estimates, based on the direct and indirect estimation methods (27, 37).

Maternal retrospective birth history data are most commonly used for child mortality indirect estimation in situations where complete vital registration data are unavailable (9). Previous empirical investigations into child mortality in LMICs relied heavily on maternal birth history data (in the forms of full or summary birth histories) to generate child mortality estimates (8), meaning that the birth-history based indirect estimation approach lacks “ground truth” data that could be use to validate the estimates. However, it is not easy, especially for male data collectors to talk with women about child birth issues in the PNG local context, where asking women about their sexual reproductive behaviours is considered as insulting (38). This challenges population-based studies and surveys conducted in PNG using face-to-face interview data collection strategies. In this study, the large vital statistics data from households were therefore used for the first time for the direct estimation of child mortality indicators at the sub-national level in PNG.

Comparing the results of the direct and indirect estimations, we see that similar IMR estimates for 2014 were produced, 53 and 67 child deaths per 1,000 live births, respectively. Similarly, the U5MR estimates for 2014 were 102 and 105 per 1,000 live births for the direct and indirect estimates, respectively. These findings suggest that the indirect estimates are generally consistent with direct estimates. The differentials in child mortality estimates of the two methods were reconfirmed by the analysis of the means of relative differences between the direct and indirect U5MR and IMR estimates. The consistency between IMR and U5MR estimates produced by the two methods indicates indirect estimation may be useful in instances were direct estimation is not possible. The research methods used in this study have provided insights into the quality of the data produced by the PNGIMR's iHDSS.

Both direct and indirect estimations can suffer from data errors. Life table used in indirect estimation provides probabilities of deaths, based on the mortality experience among a real cohort of children (39). Indirect child mortality estimates are often biassed when women do not know their age or the ages of their children. This issue is commonly observed in the surveillance sites, where the proportion of the population having a birth or death certificate is low, particularly among children who live in rural areas (11). The biases could be even greater when the indirect estimates are drawn out from the self-reported number of children ever born and the number of children born alive but who later died. In our study, the questionnaire for women aged 15–49 contained a module on child mortality, which was specifically designed to collect data for mortality estimation. However, in some cases stillbirths and live births could have been included in women's responses to the question of how many children were ever born, leading to overestimates of child mortality. Indirect estimations could also be biassed due to high child mortality from HIV/AIDS (40), which might have led to different mortality patterns among PNG children. Data for indirect estimation of child mortality in PNG could be obtained from the national census and demographic and health surveys, which are conducted around every 10 years. Although data from these sources are not always up-to-date and are sometimes inadequate for child mortality estimation (22), they are still an option in a low resource setting like PNG, where the civil registration and vital statistics systems are often deficient (41).

The direct estimation method requires birth data from specifically designed data collection tools such as the household update book used in the iHDSS. The reliability of direct estimation of child morality depends on the correct reporting of age at death, and especially the information on date of birth and date of death, including day, month and year because this information is used to calculate the age at death of the deceased child. Missing information or misreporting of these data could have resulted in biases in direct estimations, either underestimated or overestimated. From our field observations, underreporting of child mortality is more likely to happen than over-reporting in the surveillance population.

By defining the child mortality estimations in relation to the PNG context and the availability of data, the current study contributes to the existing literature on child mortality estimation. The comparison of child mortality direct and indirect estimation methods in the local context of PNG has highlighted possible avenues to further improve the accuracy of child mortality estimates in LMICs. Both direct and indirect estimation methods work well in the PNG context, suggesting both can be replicated in low resourced settings.

The question remains as to which estimation method is a better choice to provide optimal output in low resource settings, where it is not possible to carry out both estimation methods. Comparison of mean relative differences is a common method which is used to demonstrate the differentials in estimates produced by different estimation approaches. In our study, we analysed the mean relative difference between IMR estimates provided by the direct and indirect estimations. We also used this method to examine the difference between direct and indirect U5MR estimates. Measuring mean relative differences is an approach to assessing consistency between direct and indirect estimations of a child mortality rate. For IMR estimates, the mean relative differences were 22.5% for Central, 24.2% for Eastern Highlands, 18.6% for Madang, and 23.9% for all provinces. By contrast, the mean relative differences between direct and indirect U5MR estimates were 6% for Central, 7% for Eastern Highlands, 3% for Madang, and 3% for all provinces. As shown in Table 5, compared with U5MR estimates, larger differentials were consistently observed for all IMR estimates, suggesting that the IMR estimation is more susceptible to biases than the U5MR estimation. As such, the U5MR estimates are, to some extent, more reliable than the IMR estimates. For U5MR, the difference between the direct and indirect estimates was low (3%), meaning the both direct and indirection estimations can be used depends on the availability of data and technical expertise, but cheaper method could be preferred. By contrast, the difference between IMR estimates was relatively high (24%). We cannot conclude which estimation method is more reliable than the other by comparing the mean relative differences only. Hence the IMR estimates based the iHDSS data should be triangulated with other data sources available in the country.

These data observations are important for improvement in child mortality estimation in LMICs. First for collecting birth and death records, and household socioeconomic data, the network of village-based data reporters, including both males and females is crucial. By having their own social networks including families, relatives, neighbours and friends who live in the communities, data reporters can visit households on a regular basis and they have insightful knowledge of changes in their communities. With the local background and knowledge of the surveillance sites, data collectors can provide reliable and up-to-date information on births and deaths that have occurred in their villages. This village-based data collection approach makes the iHDSS a unique data source for the monitoring and reporting of child mortality at the sub-national level, where surveillance data is likely more complete and up-to-date than the cross-sectional survey data (9, 17). However, to sustain the village-based surveillance system is expensive as it requires continuous training and supervision of the data collectors as the data collection is on-going over a long period of time.

Second, the accurate and complete birth and death data such as date of birth and date of death (including the day, month, and year) among children are crucial in providing quality data for the direct estimation of child mortality. On the other hand, women's birth history retrospective data particularly among women aged 15–19 and 30–34 years are essential for the indirect estimation of child mortality. To produce reliable estimates, recall biases and record errors could be minimised during household interviews by recruiting interviewers, who are familiar with the local contexts.

### Understanding the Variation of Child Mortality Estimates

Globally, two main factors increasing child mortality include high total fertility rates (TFR) and high incidences of preventable childhood communicable diseases (13). Noticeably, there has been little variation in TFRamong woman of reproductive age in PNG over the period 2006–2016. Furthermore, the proportion of vaccination coverage among children aged 12–23 months in PNG dropped by half, from 52 to 20% in the same period. As they are the key factors influencing child mortality, one can expect little improvement in child mortality indicators at the national level in the period 2000–2020's.

The surveillance data used in this study focused only on rural areas, where child mortality is higher than urban areas due to its lower socioeconomic development status. Children living in rural areas often experience higher mortality risks than their urban counterparts due to limited access to health services and lower education of parents (42). Central Province appears to be exceptional among the four provinces with consistently lowest estimates across all three child mortality indicators over the period 2014–2017. It is possibly because this province has higher socioeconomic development than other provinces. Hiri surveillance site in Central Province is located surrounding the PNG Liquefied Natural Gas (LNG) Project, where urbanisation is occurring at a rapid pace, resulting in a marked improvement in the transportation infrastructure in the province. The population in Central Province have better access to public services such as electricity, water supply, and education and healthcare services (43).

The declining trend in child mortality was observed across the surveillance sites in the period 2014-2017 (see Table 3). For example, U5MR for all provinces declined from 100 child deaths in 2014 to 90 in 2015, 86 in 2016 and 2017. The data also highlighted the variation in the child mortality estimates across provinces. Hence, understanding local settings and contextual factors underlying the variations in child mortality estimates at the sub-national levels is important knowledge that would be helpful for the development of effective interventions, and monitoring and evaluation of the implementation of public health programs at the local level.

The declining trend in child mortality in EHP was shown as the U5MR estimate declined from 150 in 2014 to 120 in 2015, 110 in 2016. This trend was even more obvious in Hela Province, with U5MR declining from 150 in 2015 to 140 in 2016 and 120 in 2017. However, such high U5MR in the highlands region is of great concern. Previous studies showed that the highlands region was well known for high child mortality in the past, and Eastern Highlands and Hela provinces had historically higher mortalities than the national level in the 1990's (31, 44). The possible factors underlying the high level of U5MR observed in these provinces need to be further investigated. From our field observation, the high U5MR in these provinces might be associated with a measles outbreak in the highlands region between 2014 and 2017. Measles is a highly infectious disease and one of the leading causes of deaths among CU5 in developing countries and globally (32, 33). A study of the global causes of child mortality showed that preventive measures for childhood infectious diseases have halved the 3.6 million child deaths recorded in the period 2000–2013 (13). In PNG, the measles outbreak was first reported in Eastern Highlands and Hela in the 1990's (34). Since then a series of measles outbreaks have been reported in PNG among other South Pacific countries (35). The most recent measles outbreak in PNG was reported by the iHDSS in 2014 and it was most severe in the highlands (36).

To better understand the impact of the measles outbreak on child mortality in the highlands region, we conducted further analysis of morbidity data and immunisation data available from the iHDSS in the period 2014–2017. The morbidity surveillance data from primary health facilities suggest that the outbreak possibly started in Eastern Highlands with the first measles suspected child patient recorded in 2013 (45). The outbreak scaled up and reached its peak in 2014, and declined in the following years. In Hela, the measles outbreak likely started later in 2014, reached its peak in 2015 and 2016, before it declined toward the end of 2017 (45, 46). The total caseload of under-five-years old patients presenting at the primary healthcare facilities in EHP and Hela provinces increased eight and four times in the period 2014–2015, respectively (46).

Measles vaccination coverage in the iHDSS surveillance sites was as low as 50% among children under 5 years of age (45), 70% among child patients seeking healthcare services (46), and <50% among children aged 12–23 months. Measles vaccination coverage was even lower in EHP and Hela Province, only 45 and 44%, respectively (47). These data evidence suggests that the high U5MR estimates in the highlands region i.e. Eastern Highlands (147 per 1000 live births) and Hela (127 per 1,000 live births) are likely associated with limited access to immunisation service. It is noted that measles had little impact on U5MR in Central Province (41 per 1,000 live births), with no measles cases reported in the period 2014–2015.

Epidemiologically, the measles death rate has a greater impact on U5MR, and less on IMR and NMR because children aged 0–11 months are often highly protected from measles because of immunity gained through breastfeeding (48). This paper has provided evidence of how the measles outbreaks could have contributed to an increased U5MR at the sub-national level, which was hidden in the national data. Lessons learned from vaccination programmes in LMICs suggest that inequity in socioeconomic development are the root causes of the ineffective delivery of the immunisation service (30). The reduction of U5MR will depend on addressing the most common causes of death such as childhood communicable diseases (49). More efforts including supplementary and regular immunisation programmes are needed to improve the vaccination coverage among CU5 (50).

National Censuses and DHS are the main data sources of child mortality estimation in PNG. These cross-sectional data are collected every ten years, with the most recent National Census conducted in 2010 and the DHS in 2016. Key population health indicators derived from these data sources are presented in Table 6. According to these data sources, U5MR estimates halved over the past three decades, from 90 deaths per 1,000 live births in the 1990's to 80 per 1,000 live births in the 2000's and 50 per 1,000 live births in the 2020s (51). The IMR estimates were also halved from 70 child deaths per 1,000 live births in the 1990's to 30 in the 2020's. These data sources provided U5MR estimates at the national level, but not on the variations of U5MR across geographical regions or between provinces. These data sources failed to provide estimates of IMR and NMR in the period 1990–2010. The 2020 National Census was launched on 17 August 2019, but no data has been released. The estimation of U5MR from this data source needs to be closely followed up.

**Table 6:** Key population health indicators of PNG, national statistics office's national censuses and demographic health surveys, 1990–2020's.

	**Census 1990**	**DHS 1996**	**Census 2000**	**DHS 2006**	**Census 2010**	**DHS 2016**
Under 5 mortality rate (per 1,000 live births)	91.6	92.8	80.5	74 0.7	63.9	49.0
Infant mortality rate (per 1,000 live births)	N/A	69.3	N/A	56.7	N/A	33.0
Neonatal mortality rate (per 1,000 live births)	N/A	31.6	N/A	29.1	N/A	20.0
Total Fertility Rate (number of children per women)	N/A	4.8	N/A	4.4	N/A	4.2
Vaccination coverage (children aged <12 months)	N/A	38.7%	55%	52.1%	61%	20%

**Figure 2:**
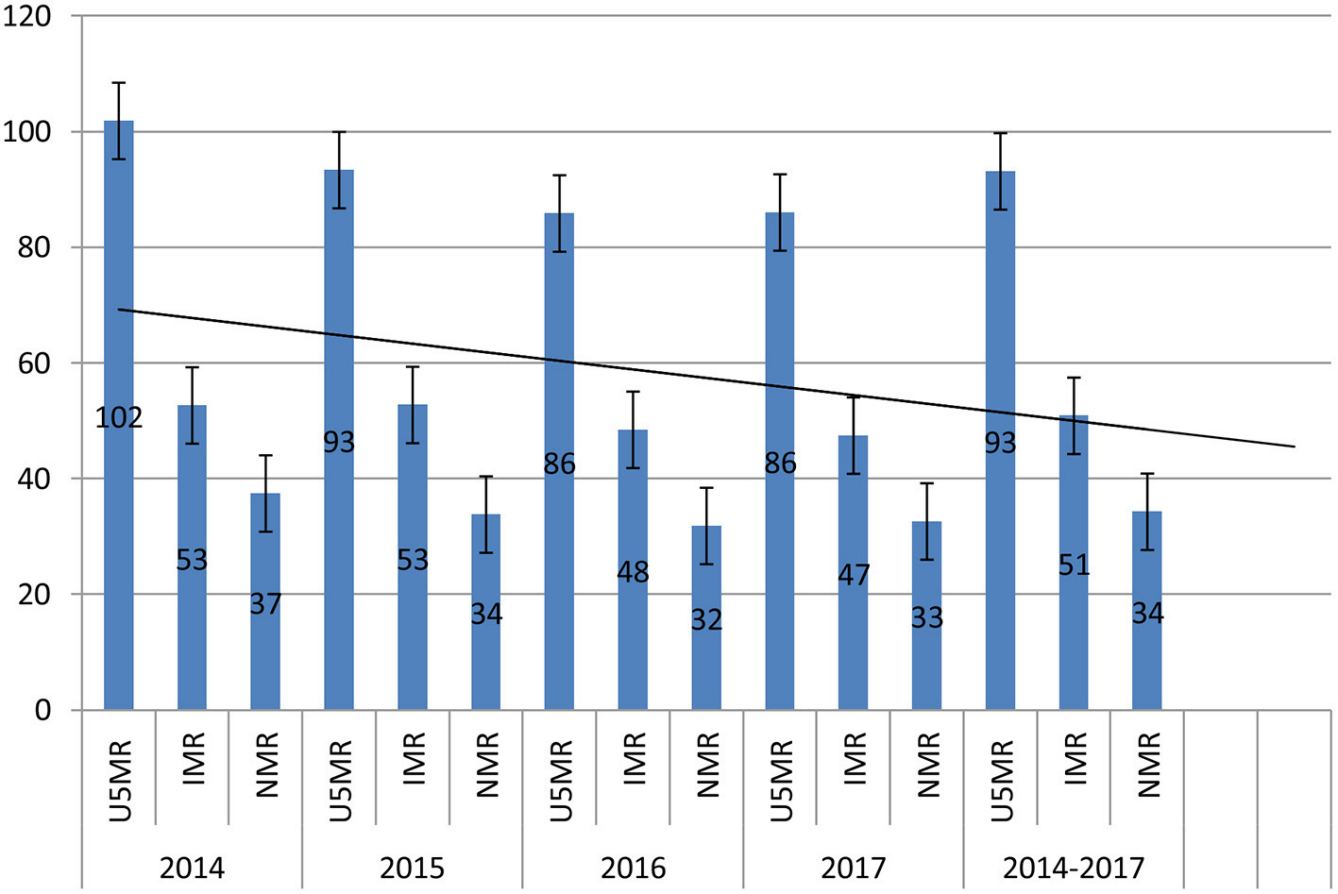
Under 5 mortality rate, infant mortality rate and neonatal mortality rate, with error bars and overall forecast linear trend line, PNGIMR's iHDSS, 2014-2017.

Compared to the national data, the sub-national child mortality estimates presented in this study are higher. This could be because the iHDSS data had captured local contextual factors such as women fertility, childhood communicable diseases and immunisation service. As shown in Figure 2, our data suggests an overall trend of declining child mortality at the sub-national level in PNG in the period 2014–2017. This trend is likely to be sustained in the coming years. However, it is unlikely to meet the national targets for child mortality as set out in the MTDP 2018–2022. The above findings show the gaps in data for child mortality analysis, strongly suggesting a pressing need for closely monitoring and reporting child mortality at the national and sub-national levels. More concerted efforts are required to further examine child mortality at the sub-national level. Data sources other than DHS and National Censuses available in the locality such as the iHDSS should be used for the estimation of child morality at the sub-national level, thus strengthening reliability of child mortality estimates in PNG.

### Limitations

There are a number of limitations in the surveillance data used for estimations of child mortality in PNG. The PNGIMR's iHDSS provides a data series for the estimation of U5MR, IMR and NMR only at the sub-national level. The iHDSS is an important additional data source for monitoring and reporting child mortality in the rural areas of PNG, it did not offer disaggregated data for rural and urban sectors as of 2017, which is now a requirement for the PNG Government in the implementation of the SDG and reporting child mortality indicators (52). This limitation has been fixed since early 2018, when the iHDSS was upgraded to a comprehensive health and epidemiological surveillance system (CHESS) (53).

The selection of the four iHDSS sites was purposively based on the consultations and consensus with stakeholders at the national and provincial level. These sites were not representative for the provinces where they are located, in terms of socioeconomic development. Hiri and Hides were two sites experiencing considerable socioeconomic and demographic changes associated with the implementation of the PNG Liquefied Natural Gas Project in early 2010's. By contrast, Asaro and Karkar sites were selected as a continuity of PNGIMR's surveillance activities in the previous phase.

Child mortality data were collected from the populations living within the catchment areas of the iHDSS over the reporting periods. Consequently, the estimated IMR and U5MR did not necessarily represent for the mortality profile of entire child population of each province. Although child deaths in the surveillance sites were identified by data reporters, who were based in their villages and as such, were able to conduct regular household visits as part of the study on population census and demographic change, there was no guarantee that all the child deaths had been adequately reported within the time frames. For these reasons, interpretations of the IMR and U5MR estimates in this study should be made with caution.

Reducing child mortality holds the key for PNG. As a member of the United Nations, in 2020 the PNG Government should be able to submit a report for the first 5 years of the implementation of SDGs. More effort, national resources and international assistance are therefore, needed to further accelerate the country's progress towards achieving SDG targets on child mortality by 2030. The integrated approach to collecting and reporting data in the iHDSS has made it an important data source for estimations of child mortality at the sub-national level (24). The iHDSS can provide longitudinal data series which can be used for the projection of child mortality trends in the long term. This is important infrastructure for conducting population-based epidemiological studies, clinical trials and delivery of healthcare services to the population, which are needed for the development of evidence-based and informed policy for the target populations, and to measure changes over long periods of time (54). This approach is particularly important in the global and local contexts of COVID-19 outbreaks because the local network of the iHDSS would be used to effectively roll out contact chasing, public awareness, and vaccine delivery.

Data used in this study were based on self-reported information on children's date of birth and dates of deaths. Without supporting documents such as birth certificate and death certificate, it could have led to errors in the calculation of the children's age at death (in year, month and day), which could be a bias in estimating child mortality rates. The retrospective birth history data could also have errors associated with recall biases, particularly among the older women. The Brass indirect estimation method has also limitations. A key assumption of the Brass method is the constant fertility in the recent past years, resulting from the simplified life table model (29). Although the fertility of PNG was reportedly declined in the period 2006–2016, and the decline is relatively small at the national level, but this could have varied substantially at the sub-national level. Hence, this fixed fertility assumption may result in systematic bias, potentially increasing indirect estimates of child mortality.

## Conclusion

This study has provided a systematic assessment of child morality estimates in PNG. For the first time, both the direct and indirect estimation methods were conducted using vital statistics and maternal birth history data available from the PNGIMR's surveillance system. The differential of child mortality estimates produced by the two methods was evaluated. Variation of child mortality estimates across the provinces was assessed. PNGIMR's surveillance systems are a powerful public health tool for the PNG Government agencies to monitor the child mortality at the sub-national level. These data are essential for the health sector to respond to emerging child health issues in a timely manner. The surveillance system is more important than ever in the context of COVID-19 pandemic in PNG and globally. More consultation among PNG Government and stakeholders are needed to develop a suitable and sustainable mechanism and process for integrating recent improvements in surveillance data collection such as CHESS into the routine monitoring and evaluation systems in PNG. Benefits of the system are not limited to the provision of data for health and development, but more importantly it would be a reliable referral data source, complimentary to the existing national data sources for monitoring the implementation of the international and national development agendas, including the United Nation's SDGs 2030 and the PNG's Vision 2050.

## Data Availability Statement

The raw data supporting the conclusions of this article will be made available by the authors, without undue reservation.

## Ethics Statement

The iHDSS was granted ethics approvals from Institutional Review Board of PNG Institute of Medical Research (IRB Approval No. 11.13) and the Medical Research Advisory Committee of Papua New Guinea (MRAC Approval No. 11.20). These approvals covered all the data components under the iHDSS, including data of women of reproductive health (15-49 years) and household demographic changes, which were used in this manuscript. Informed consent was sought from self-identified household heads and female participants. Women were informed about their right to withdraw from the study at any stage. Written informed consent to participate in this study was provided by the participants' legal guardian/next of kin.

## Author Contributions

BP designed and oversight the iHDSS, conceptualised the manuscript, analysed the data, drafted, revised, and finalised the manuscript. RE supervised the fieldwork, collected and analysed the data, and drafted the manuscript. TH, A-MP, and AO reviewed, provided comments, and inputs and edited the manuscript. All authors contributed to the article and approved the submitted version.

## Funding

The iHDSS operated under the Partnership in Health Project with financial support from PNG LNG Project. The funder had no role in study design, data collection and analysis, or writing of the manuscript.

## Conflict of Interest

The authors declare that the research was conducted in the absence of any commercial or financial relationships that could be construed as a potential conflict of interest.

## Publisher's Note

All claims expressed in this article are solely those of the authors and do not necessarily represent those of their affiliated organizations, or those of the publisher, the editors and the reviewers. Any product that may be evaluated in this article, or claim that may be made by its manufacturer, is not guaranteed or endorsed by the publisher.
